# The Impact of Allicin on the Growth of *Clostridium* spp. in the Digestive Track of Quails

**DOI:** 10.3390/ani15070906

**Published:** 2025-03-21

**Authors:** Aleksandra Makuch, Monika Ziomek, Magdalena Sapała, Kamil Drabik, Justyna Batkowska, Piotr Domaradzki, Ewelina Patyra, Tomasz Grenda

**Affiliations:** 1 National Veterinary Research Institute, Partyzantów 57, 24-100 Pulawy, Poland; magdalena.sapala@piwet.pulawy.pl (M.S.); ewelina.patyra@piwet.pulawy.pl (E.P.); 2 Department of Food Hygiene of Animal Origin, University of Life Sciences in Lublin, Akademicka 13, 20-950 Lublin, Poland; monika.ziomek@up.lublin.pl; 3 Institute of Biological Basis of Animal Production, University of Life Sciences in Lublin, Akademicka 13, 20-950 Lublin, Poland; kamil.drabik@up.lublin.pl (K.D.); justyna.batkowska@up.lublin.pl (J.B.); 4 Department of Commodity Science and Animal Raw Materials Processing, University of Life Sciences in Lublin, Akademicka 13, 20-950 Lublin, Poland; piotr.domaradzki@up.lublin.pl

**Keywords:** *Clostridium botulinum*, *Clostridium perfringens*, *Clostridium* spp., allicin, polymerase chain reaction, microbiological contamination

## Abstract

Allicin is a chemically complex bioactive compound synthesized in many varieties of garlic. The wide range of biological properties of allicin provides the basis for its potential use as an alternative to antibiotic growth promoters that are currently prohibited in farm animal breeding. Among the many benefits resulting from the use of allicin in animal breeding, especially poultry, its modulating effect on intestinal microbiota, which includes the anaerobic spore-forming bacteria of the genus *Clostridium* spp., seems to be important. The material for this study consists of intestinal content collected from the caecums of Japanese quails (*Coturnix japonica*). Culture methods were used to isolate the strains, and the obtained isolates were identified based on their phenotypic characteristics. In addition, PCR methods were used for the detection of the *ntnh* gene-encoding non-haemagglutinin component of botulinum neurotoxins (BoNTs), the detection of individual genes responsible for the production of major toxins by *Clostridium perfringens*, and the amplification of conservative 16S rDNA genes. The 16S rDNA amplicons were subsequently submitted to Sanger sequencing. The obtained sequences were analyzed using the Basic Local Alignment Search Tool (BLAST). The *ntnh* gene was not found in the genetic material of the isolated strains. Among the isolates suspected of belonging to the *Clostridium perfringens* species, the *plc* gene determining the production of the alpha toxin was detected, which justifies the classification of the strains into toxotype A. The Sanger sequencing results confirm the presence of mainly saprophytic species in the studied material. The statistical analysis indicated a statistically significant reduction in the level of *Clostridium* spp., obtained by the use of an appropriate dose of allicin. The presented research results indicate the significant impact of an appropriate dose of allicin on reducing the occurrence of anaerobic intestinal microbiota, while providing important information on the potential application of this compound in animal production in the future.

## Simple Summary

Allicin, as a compound naturally occurring in garlic, has well-known hypocholesterolemic and antimicrobial properties that have been known for a long time. A broad range of biological properties allows for the potential use of this compound in animal production. The aim of this study is to determine the effect of allicin on the growth of anaerobic microbiota of the *Clostridium* spp. isolated from the digestive tracts of Japanese quails and also the molecular characteristics of isolated strains. Based on the results of this study, a statistical trend is observed, regarding the effect of allicin at a dose of 200 µg/kg, showing a decrease in *Clostridium* spp. contamination. Statistical analysis showed a significant reduction in *Clostridium* levels after allicin was fed to animals at a dose of 250 µg/kg. The results of this study show that allicin supplied to animals in an appropriate dose not only reduces the level of *Clostridium* spp. in the digestive tract of animals in a statistically significant manner, but may also have a beneficial effect on modulating the species composition of the intestinal microbiome. Taking into account the results obtained and the small amount of literature data available on this subject, there is a need for further research on the possible use of allicin in animal nutrition.

## 1. Introduction

Improving consumer awareness of the effects of antibiotic residues in animal products and the European Union’s ban on the subtherapeutic use of antibiotic growth promoters (AGPs) have contributed to an intensive search for alternative substances whose use in the animal industry would not adversely affect animal productivity and the quality of the products obtained. The currently published literature data indicate considerable interest in the potential use of allicin for this purpose, a bioactive sulfur compound found naturally in many varieties of garlic [1,2,3].

The targeted use of allicin as an agent with antimicrobial activity has been known for a long time [3]. The precursor necessary for the synthesis of this compound is the non-proteinogenic amino acid alliin, which, like other S-alkyl-L-cysteine sulfoxides, is hydrolyzed by a vacuolar enzyme called alliinase. Both alliin and the aforementioned enzyme are found in separate fragments of the garlic clove, so the enzymatic transformation of the precursor is initiated only by damage to the plant tissue [4]. An enzymatic reaction occurring in the presence of water produces an unstable complex of alliin and allinase, which then undergoes dehydration assisted by pyridoxal phosphate. The occurring reaction leads to the formation of dehydroalanine, as well as unstable and very active at room temperature allilosulfenic acid [4,5]. At room temperature, the resulting allylsulfenic acid molecules spontaneously condense to form an allicin molecule [6]. The compound rapidly decomposes into other organosulfur compounds that are readily soluble in water and oil, such as diallyl disulfide, diallyl trisulfide, and diallyl sulfide, and shows high reactivity with the thiol groups of many proteins, to which it owes its antimicrobial activity [7].

Currently, the use of allicin in livestock farming is not practiced. However, the use of this bioactive substance in animal production may be particularly advantageous due to the low costs associated with its use, as well as the possibility of obtaining an active ingredient characterized by high purity and significant medicinal properties [1,8]. The particular benefits of allicin have been observed on poultry farms, where the effect of this compound on improving animal growth parameters, resulting from increased feed intake and, consequently, increased weight gain, has been documented. However, the detailed mechanisms responsible for the resulting improvements in poultry weight parameters do not remain completely elucidated. According to some researchers, the addition of garlic extract containing allicin to feed can stimulate the appetite of animals, translating into a voluntary increase in food intake and weight gain [9,10]. Based on the results of their study, Kirubakaran et al. [11] interpreted the observed weight gain in poultry as an effect of the substances contained in garlic, which can stimulate faster saliva flow and increased gastric juice secretion in poultry, which may consequently result in improved digestive processes occurring in the animals’ bodies. However, a number of studies have also reported the ineffectiveness of allicin and the lack of any apparent effects of supplementation in poultry, and the resulting variability in the effectiveness of allicin on animal weight gain may depend on the quality of the product used, its dosage, as well as the duration of supplementation [10].

The use of allicin in animal production also refers to the use of this compound as a hypolipidemic and hypocholesterolemic agent, as well as an agent to improve the quality of animal products, especially poultry eggs. Studies have shown that allicin supplementation in feed can favorably affect the fatty acid profile of egg yolk by increasing the level of polyunsaturated fatty acids [1,8,12]. Some studies also report the possibility of using allicin to modulate the intestinal microbiome [13].

The gastrointestinal tract of animals and humans is mainly inhabited by commensal microorganisms, belonging to eukaryotes, viruses, and bacteria. Significant recent advances, especially in the field of metagenomics and next-generation sequencing, have made it possible to identify four types of bacteria that make up the vast majority of the intestinal microbiota [14]. It has been shown that there are three groups of anaerobic extremophilic bacteria among the listed microorganisms, as follows: the *Clostridium coccoides* group (forming cluster XIVa), within which there are 21 species of bacteria; the *Bacteroides* and the *Clostridium leptum* group (forming cluster IV), with 4 species of microorganisms; and it is also believed that bacteria belonging to the listed groups of the genus *Clostridium* spp. are an important part of the intestinal microbiota [15,16,17]. The genus *Clostridium* spp. is represented by Gram-positive anaerobic bacteria capable of producing spores and characterized by high heterogeneity within strains. The natural reservoir of these microorganisms is soil, marine sediments, and the large intestine, particularly the mucosal folds of the ascending colon, where these bacteria are characterized by their ability to produce short-chain fatty acids, which have an important function in intestinal homeostasis processes [16]. Bacteria belonging to the genus *Clostridium* spp. are mostly described as a biohazard carrying huge economic losses and contributing to the loss of human and animal health or life; however, these microorganisms have also found applications in many industries such as textiles and in the production of solvents. In addition, there are strains with significant medical significance, so that they can be used in various therapies beneficial to animal and human health. *Clostridium* spp. are also characterized by a high diversity of metabolic functions, including the ability to convert starches and proteins into alcohols, organic acids, hydrogen, or carbon dioxide [18].

The first aim of this study was to determine the effect of allicin on the growth of anaerobic microbiota of the genus *Clostridium* spp. isolated from the digestive tracts of Japanese quails. Additionally, this study aimed at the molecular characterization of the obtained isolates, showing phenotypic similarity to the pathogenic species such as *Clostridium botulinum* and *Clostridium perfringens*. Also, the characterization of *Clostridium* isolates was conducted by 16S rDNA.

## 2. Materials and Methods

### 2.1. Sample Collections

This study was approved by the local ethical committee for animal experiments at the University of Life Sciences in Lublin (approval no. 35/2021). The period for conducting the experiments was the years 2023/2024. The material for the study consisted of cecums taken from Japanese Pharaoh quails kept under experimental conditions. Quails during experimental rearing were divided into 4 groups (labeled 1G to 4G). Within each group, 8 replicate subgroups (cages) of 16 birds per group were separated (64 birds/all groups). The caecum of quails from each cage were treated as a pooled laboratory samples. In total, 32 caecum samples were subjected to further microbiological examinations towards detection of *Clostridium* spp. The birds were maintained in 0.5 m^2^ cages placed on the same level, positioned side-by-side along the long walls of the building. During the experiment, a standard photoperiod (17 h of light:7 h of darkness), continuous ventilation and an optimal temperature of 18 °C were maintained. The quails had unlimited access to water and feed. During rearing, the typical feed mixture was used. Detailed information can be found in File S1 of the Supplementary Materials. The experiment was started at the 7th week of birds age, according to their sexual maturity and the beginning of egg production [19]. The experimental factor was the supplementation with the aqueous solution of standardized allicin to drinking water in the following doses: G1 (control group)—no addition of allicin in the water, G2 (experimental group 2)—received a dose of 150 μg of allicin per kg of body weight, G3—200 μg/kg, and G4—250 μg/kg. The allicin preparation was purchased from GrupamediaM.pl sp. z o.o. (Łódź, Poland) in the form of spray-dried allicin and freeze-dried alliinase enzyme, manufactured according to the polish patent No. P.442206. The allicin was prepared according to the manufacturer’s instructions. Briefly, alliin was dissolved in water (2.4% *w*/*v*) and alliinase enzyme was dispersed in water (0.2% *w*/*v*). Afterward, both solutions were mixed in a ratio of (1:1) and incubated at room temperature for 20 min. The allicin solution was prepared immediately before administration (*m*/*v*) and then dispensed directly into manually filled drinkers, with the amount of test substance and the amount of water determined based on the birds’ current body weight and water intake, so that the birds would take the required dose while ensuring optimal drinking water. The allicin was applied at 3-day intervals for 12 weeks. At the end of experiment, 64 birds were slaughtered in accordance with Council Regulation (EC) No 1099/2009 of 24 September 2009 on the protection of animals at the time of killing by dislocation of the cervical vertebrae [19].

### 2.2. Cultures

The caecum samples were inoculated at 1 g into Tarrozi-Wrzosek liquid medium (1 L liver extract, 10 g/L peptone, 5 g/L sodium chloride, 5 g/L glucose, 3–4 beef liver cubes) enabling culture of anaerobic bacteria. To determine the level of contamination with *Clostridium* bacteria, serial decimal dilutions (1:10, 1:100, 1:1000, etc.) of the analyzed samples were prepared in accordance with PN-R-64791:1994 [20] by inoculating 1 mL each of the test material in duplicate into tubes containing Tarrozi-Wrzosek medium. The level of contamination was adequate to the dilution at which Clostridia growth was observed. The results were expressed as log (cfu/g). Inoculated samples from one replicate were heat-treated at 70 °C for 15 min. The inoculum prepared in this way was incubated at 37 °C for 48 h, with anaerobic conditions obtained using an anaerobic incubation system including appropriate anaerobic jars and anaerobic atmosphere-generating sachets (AnaeroGen, Thermo Fisher Scientific, Waltham, MA, USA), as well as test strips to detect an anaerobic atmosphere (Merck Millipore, Darmstadt, Germany). After the incubation period, anaerobic growth was observed on the liquid medium.

In the next step, 10 µL of liquid cultures were taken from the tubes and spread on Willis–Hobbs differentiation agar (10 g/L peptic digest of animal tissue, 10 g/L meat extract, 5 g/L sodium chloride, 12 g/L lactose, 0.032 g/L neutral red, 10 g/L skim milk powder, 2 g egg yolk powder, and 10 g/L agar, with a final pH of 7.0 ± 0.2 at 25 °C). The prepared plates were incubated under anaerobic conditions at 37 °C for 48 h. The obtained colonies were evaluated for their shape, surface area, size, and proteolytic, lipolytic, or lecithinolytic features.

### 2.3. Detection of the Ntnh Gene Using Real-Time PCR

The genetic material used in the PCR reactions was isolated from 1 mL liquid cultures and a few selected colonies obtained from agar plates. DNA was extracted using a commercial Genomic Mini AX Bacteria kit (A&A Biotechnology, Gdynia, Poland) according to the manufacturer’s instructions. The amount of DNA used in the PCR reaction varied from 1 to 25 ng. The amount of DNA was estimated using a Nicolet Evolution 300 spectrophotometer (Thermo Fisher Scientific, Waltham, MA, USA). The extracted DNA was frozen at −20 °C or directly analyzed using PCR.

The *ntnh* gene is responsible for determining the production of the non-toxic non-hemagglutinin component of botulinum neurotoxin and is a characteristic element in the botulinum gene cluster for all strains of the genus *Clostridium* with the ability to produce botulinum toxin. To detect the *ntnh* gene, a set of seven primers and a Taqman probe were used according to the methodology described by Raphael and Andreadis [21]. The sequences of primers and probe used to carry out the PCR reaction are included to the Supplementary Materials, File S2. The reaction was performed using the following reagents: 5 μL DNA, 4 μL LightCycler TaqMan Master (Roche, Basel, Switzerland), 0.7 μM of each primer, and 0.24 μM of NTNH410 TaqMan probe. The real-time PCR was performed on a LightCycler 2.0 thermocycler (Roche, Basel, Switzerland) with the following thermal cycling profile: 10 min at 95 °C as initial denaturation and 40 cycles of denaturation at 95 °C for15 s, annealing at 42 °C for 15 s, and elongation at 55 °C for 1 min. The fluorescence signal was captured at the elongation step in each cycle. As the control samples, DNA isolated form the following reference strains was used: *C. botulinum* NCTC 887, *C. botulinum* NCTC 3815, *C. botulinum* NCTC 8266, and *C. botulinum* NCTC 10281.

### 2.4. Clostridium Perfringens Detection

The strains isolated on agar medium suspected to belong to the *Clostridium perfringens* species were analyzed using multiplex PCR performed according to the methodology described by Rood et al. [22] using a temperature profile involving initial denaturation at 95 °C for 5 min and 35 cycles consisting of denaturation at 95 °C for 1 min, annealing at 55 °C for 1 min, and elongation at 72 °C for 1 min. The sequences of primers used to carry out the reactions are included in the Supplementary Materials. This method allows for simultaneous detection on a 2% agarose gel of *plc*, *cpb*, *etx*, *iap*, *cpe*, and *netB* genes, from which it is possible to determine the toxic type of this microorganism. The *C. perfringens* ATCC 13124 strain was used as a positive control in the reaction.

### 2.5. Detection of Clostridium Strains Using Amplification and Sanger Sequencing of 16S rDNA

Unidentified strains suspected to belong to the genus *Clostridium* spp. were subjected to identification on the species level performed by amplification of the conserved 16S rDNA gene according to the methodology described by Vaneechoutte et al. [23]. The sequences of primes used to carry out the PCR reactions are included in the Supplementary Materials. The reaction volume was 25 μL, and the reagent components were 5 μL of DNA template, 2.5 μL of 10 × Taq buffer with KCl (Thermo Fisher Scientific, Waltham, MA, USA), 4 mM MgCl2, 200 μM dNTPs, 0.3 μM of each primer and 1.25 U/25 μL of Taq polymerase (Thermo Fisher Scientific). The reaction was carried out on a Biometra T1 thermocycler (Biometra, Gottingen, Germany) as follows: initial denaturation at 95 °C for 5 min, followed by 35 cycles of denaturation at 95 °C for 45 s, annealing at 55 °C for 45 s, and elongation at 72 °C for 1 min. The cycling step was followed by final-strand elongation at 72 °C for 10 min. The obtained length of the amplicons was about 1500 bp. The sequence analysis of the PCR products was outsourced to Genomed (Warsaw, Poland). The results provided in FASTA files were analyzed using the BLAST (Basic Local Alignment Search Tool, https://blast.ncbi.nlm.nih.gov/Blast.cgi?PROGRAM=blastn&PAGE_TYPE=BlastSearch&LINK_LOC=blasthome, accessed on 12 November 2024) algorithm and compared with sequences available in the NCBI (National Center for Biotechnology Information) database.

### 2.6. Electrophoresis of PCR Products

Agarose gels were prepared at a concentration of 2% in 1× Tris-acetate-EDTA (TAE) and visualized using a SimplySafe nuclease dye (EURx, Gdansk, Poland). Electrophoretic separation was carried out for 1.5 h at 100 V. The molecular weight of the obtained products was assessed using a GeneRulerTM 100 bp DNA Ladder Plus (Thermo Fisher Scientific, Waltham, MA, USA).

### 2.7. Statistical Analysis

Statistical analysis was carried out for the results of the level of occurrence of *Clostridium* bacteria expressed as log (cfu/g). The Shapiro–Wilk test was used to determine the normality of the distribution of the obtained data for all experimental groups and the control group. Based on the results of the test, a statistical analysis of the effect of allicin content on the growth of anaerobic microbiota isolated from the digestive tracts of quails was carried out using the chi-squared test. Two null hypotheses were considered during the statistical evaluation:

1. The addition of allicin fed to quails does not significantly affect the level of *Clostridium* spp. isolated from the digestive tracts.
2. The addition of allicin fed to quails reduces the level of *Clostridium* spp. isolated from the digestive tracts.

The results were expressed as *p*-values and considered statistically significant when the *p*-value was <0.05. A *p*-value between 0.05 and 0.1 was considered to be a statistical trend.

## 3. Results

### 3.1. Level of Clostridium spp. Contamination and Statistical Analysis Results

**Figure 1:**
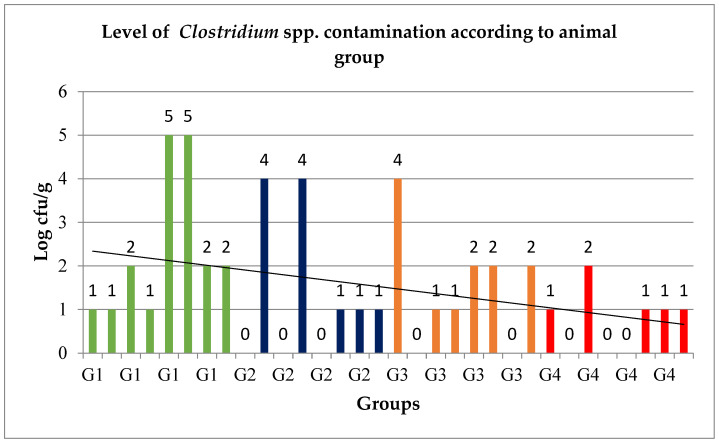
The level of *Clostridium* spp. contamination depending on the group of animals tested (details about contamination level can be found in the Supplementary Materials, File S3).

The results of determining the level of *Clostridium* spp. in the control group and in the individual experimental groups are shown in the chart below (Figure 1).

In the control group (G1), the level of *Clostridium* spp. ranged from 1 log (cfu/g) to 5 log (cfu/g), whereas in the first experimental group (G2) involving animals given 150 µg/kg allicin, the level of *Clostridium* spp. ranged from 0 log (cfu/g) to 4 log (cfu/g). In the next experimental group (G3) involving animals watered with 200 µg/kg allicin, the level of *Clostridium* spp. ranged from 0 log (cfu/g) to 2 log (cfu/g), while for the fourth group (G4), for which a dose of 250 µg/kg allicin was used, the level ranged from 0 log (cfu/g) to 1 log (cfu/g).

The obtained data on the contamination level of *Clostridium* spp. in the control group (G1) and in the individual experimental groups (G2–G4) did not have a normal distribution, which formed the basis for further analysis using a non-parametric chi-square test. Statistical analysis showed no statistical significance for the effect of allicin fed at a dose of 150 µg/kg (G2) on the level of *Clostridium* spp. contamination, as evidenced by a *p*-value of 0.93. A statistical trend was observed for the effect of allicin at a dose of 200 µg/kg (G3) on *Clostridium* spp. contamination (*p*-value = 0.68). Statistical analysis showed a significant reduction in *Clostridium* levels after allicin was fed to animals at a dose of 250 µg/kg (G4), as evidenced by a *p*-value < 0.05.

### 3.2. Identification of Isolated Strains

Among the 50 strains isolated in Willis–Hobbs agar medium, a significant number of isolates showed phenotypic similarity to *C. botulinum* species. These strains were characterized by a shiny “pearly layer” resulting from lipolytic properties that are a particularly important feature for the mentioned species. The growth of the seven strains was observed in the form of yellow, round, and convex colonies, which was the basis for their classification as strains phenotypically similar to the species *C. perfringens*.

### 3.3. Real-Time PCR Analysis Results

Real-time PCR analysis performed for genetic material extracted from liquid cultures and from phenotypically similar *C. botulinum* strains isolated from agar medium Willis–Hobbs did not reveal the presence of the *ntnh* gene. Positive analysis results were obtained only for the *C. botulinum* reference strains.

### 3.4. Multiplex PCR Results for Clostridium Perfringens Detection

Seven strains phenotypically similar to the *C. perfringens* species were isolated from the tested material. Multiplex PCR analysis revealed, for five isolates, the presence of the *plc* gene (324 bp), the presence of which formed the basis for classifying the aforementioned strains into toxotype A, which is characterized by the ability to produce alpha toxin.

### 3.5. Results of the 16S rDNA Gene Sequencing

Isolates suspected to belong to the genus *Clostridium* spp. were subjected to analysis of the conserved 16S rDNA gene. From the isolates, 21 strains were obtained whose sequences showed similarity to the *C. sporogenes* species. The percentage of similarity between the obtained amplicon sequences and those deposited at NCBI ranged from 89% to 97%. In addition, the sequencing results indicate the presence of three isolates showing similarity to *Clostridium sartagoforme* (87–95%), one to *Clostridium butyricum* (94%), and one to *Clostridium intestinale* (79%). Based on an analysis of the 16S rDNA gene, five strains were also found to have sequences similar to *Clostridium perfringens* (94–96%) and two to *Clostridium saccharolyticum*, for which the percentage of sequence similarity ranged from 94% to 95% (Table 1).

## 4. Discussion

Anaerobic spore-forming bacteria of the genus *Clostridium* spp. are an important part of the intestinal microbiota of animals and humans, where they perform important functions in maintaining normal intestinal homeostasis. However, some species of the genus *Clostridium* are capable of producing dangerous toxins, making them considered etiological agents of serious diseases. In addition, these microorganisms pose a major diagnostic challenge due to the high heterogeneity within the genus, the possibility of horizontal gene transfer occurring between strains, and the lack of suitable selective media to isolate the *Clostridium* spp. microbiota [24]. The microbiological medium used in the present study (Willis–Hobbs agar) makes it possible to observe morphological features characteristic of certain *Clostridium* species, which is particularly relevant from an epidemiological point of view.

The results of the analysis of genetic material obtained from the anaerobic intestinal microbiota of quails, carried out using real-time PCR, did not reveal the presence of the *ntnh* gene characteristic of *Clostridium* spp. strains capable of producing botulinum toxins, while it did indicate the presence of strains phenotypically similar to the pathogenic species *Clostridium botulinum*. In addition, the results of this study confirm the presence of five isolates classified based on the presence of the *plc* gene to the *Clostridium perfringens* type A species, which has the ability to produce alpha toxin. It is known that CPA was the first bacterial toxin to exhibit enzymatic properties. It is now recognized as the most important etiological agent of the highly fatal gas gangrene. The clinical symptoms of this disease include, in particular, skeletal muscle necrosis, pulmonary emphysema, and multiple organ failure, with death as a consequence [25]. Some of the literature sources describe the ability of *C. perfringens* type A to cause gastrointestinal syndromes in poultry, such as clinical necrotic enteritis; however, linking specific diseases to the mentioned toxotype can be difficult due to the opportunistic nature of this microorganism [26,27]. The natural reservoir of *Clostridium perfringens* is primarily soil and food; however, the species is also part of the microbiota of the gastrointestinal tract of animals and humans. The onset of disease symptoms due to *C. perfringens* type A species most often occurs as a result of immunosuppression that may be the result of a change in diet, a history of antibiotic therapy, and existing disturbances in the composition of the intestinal microbiota, as well as developing inflammation and the conduction of gastrointestinal surgery [26].

The results obtained from Sanger sequencing and the bioinformatics analysis performed show, among the isolates, the presence of not only *C. perfringens* species, but also isolates whose 16S rDNA sequences show significant similarity to species such as *C. sporogenes*, *C. sartagoforme*, *C. butyricum*, *C. inestinale*, and *C. saccharolyticum*. *Clostridium sporogenes* is considered to be a species with phenotypic characteristics identical to *C. botulinum*, accounting for many of the difficulties that occur when determining the affiliation of the tested strains to the pathogenic *C. botulinum* species. *C. sporogenes* usually does not have toxigenic properties and does not contribute to the symptoms of botulism, characterized by descending flaccid paralysis. However, recent reports from several years ago indicate that some strains belonging to this species can produce BoNT/B [28,29]. In addition, the proteolytic nature of *C. sporogenes* and its ability to produce spores characterized by high heat resistance make the difference between the two species appear to be very small. Some strains of *C. sporogenes* have been described in the literature as non-toxic variants of group I *C. botulinum*, an example of which is the *C. sporogenes* strain PA 3679 described by Lee and Riemanaa. In their study, the authors pointed out the 100% homology of PA 3679 to *C. botulinum* strain 62A [30].

*C. sartagoforme*, similar to *C. saccharolyticum*, is considered a saprophytic species. This microorganism has the ability to degrade cellulose, chitin, and carboxymethylcellulose. The reaction results in the production of such compounds as propionic acid, formic acid, hydrogen, and butyric acid. In addition, some of the strains of this species may also have the ability to produce N-acetylglucosaminidase, which damages the cell wall of Gram-negative bacteria. Due to the production of a number of substances that can have positive effects on animal health, some strains of *C. sartagoforme* can be considered as probiotics [31].

The species *C. intestinale* is considered to be a pathogenic microorganism that can contribute to rare bacteremia. It was first described in 1989 by Lee et al. [32]. Isolates of this species are mostly aerotolerant and saccharolytic, capable of fermenting carbohydrate compounds. Currently, there are few reports in the literature of the colonization or growth of an infection caused by *C. intestinale*. The only episode of bacteremia has been described in a human by Elsayed and Zhang [33]; however, the authors of the study suggest that the gastrointestinal tract of the patient was probably colonized before the episode of bacteremia occurred, and the factors that increase the risk of bacteremia are mainly malignant neoplasm, history of surgery, injury, and intestinal perforation.

*C. butyricum* strains, on the other hand, can occur in a variety of niches, but primarily their presence is found in soil, vegetables, and fermented dairy products, although bacteria belonging to this species also occur naturally in the digestive tracts of humans and animals [34]. This species can produce short-chain fatty acids by fermenting undigested dietary fiber, particularly butyrate, which is the main reaction product, but also acetate. Due to a number of possible positive health effects, *C. butyricum* strains can be considered as probiotics; however, some of them can also be the etiological agent of many serious pathological conditions. There are reports in the literature that indicate that *C. butyricum* can produce BoNT/E, leading to botulism symptoms [29,35].

The strains belonging to the genus *Clostridium* spp. isolated during the study are mainly commensal species and are commonly found in the digestive tracts of humans and animals. In some cases, this natural intestinal microbiota may also be the etiological agent of food poisoning. The properties possessed by some of the aforementioned species may be particularly valuable for their use as probiotics, but concerns remain about the safety of their use, especially *C. butyricum*, which can benefit intestinal health by releasing SCFAs, among other things, but which in some cases is also a pathogen that leads to serious diseases [34].

The level of *Clostridium* spp. contamination was determined in accordance with PN-R-64791:1994 [20]. The results show a statistically significant effect of the higher allicin dose on the level of *Clostridium* spp. contamination. A *p*-value ranging from 0.05 to 0.1 testified to a statistical trend regarding the effect of the 200 µg/kg allicin dose on *Clostridium* spp. contamination. The level of bacterial contamination was found to be significantly lower in the experimental group, where a dose of 250 µg/kg allicin was used to water the animals (*p*-value < 0.05). The results obtained testify to the confirmation of the null hypothesis that the level of *Clostridium* spp. isolated from the gastrointestinal tract of quails is reduced by feeding animals with the appropriate dose of allicin. The *Clostridium* spp. strains obtained during the study, isolated from the digestive tract of quails constituting the control group and the experimental group, in which a dose of 150 µg/kg allicin was applied, belonged mainly to nontoxic species, phenotypically similar to the pathogenic *C. botulinum*, but capable of acquiring traits conditioning the production of botulinum toxins as a result of the horizontal gene transfer that takes place. With increasing allicin dose, we also observed the appearance among isolates of strains with characteristics indicating their potential use as probiotics.

The antimicrobial properties of allicin have long been known. This compound can exhibit a broad spectrum of action against both Gram-positive and Gram-negative bacteria. Currently, few reports in the literature are available that describe the effect of allicin on the growth of anaerobic intestinal microbiota, which includes *Clostridium* spp. An important spectrum of action of this compound was described in their work by Ankri and Mirelman [36]. In conducting their research, the authors noted the sensitivity to allicin in various strains showing resistance to antibiotics. Among these bacteria listed were methicillin-resistant *Staphylococcus aureus*, multidrug-resistant enterotoxigenic strains of *Escherichia coli*, and *Shigella sonnei*. The positive effect of allicin was also described in the work by Jonkers et al. [37]. The authors conducted research on the inhibitory effect of this compound on the bacterium *Helicobacter pylori*, responsible for some of the most common gastrointestinal diseases. The results of the aforementioned studies show an inhibitory effect on five clinical isolates of this bacterium. Some of the ranges of action of allicin presented above suggest that this compound may play an important role in inhibiting the growth of many microorganisms that cause serious pathological conditions in both humans and animals, thus contributing to the development of new therapeutic applications.

The described results of this study suggest that allicin, when used in an appropriate dose, not only reduces the level of *Clostridium* spp., but can also affect the species composition of the anaerobic microbiota, contributing to a reduction in the occurrence of potentially pathogenic species and the appearance of species with potentially probiotic properties [16,34]. However, based on the literature data, it is noticeable that allicin is rarely used in poultry production, which may be due to the great inconsistency in the effectiveness of studies conducted so far and the lack of a complete understanding of the mechanisms of action of this compound. The main limitation of the present study is the small number of available studies describing the possible effects of allicin on the intestinal microbiota of animals, particularly of *Clostridium* spp., that can be the etiological agent of many diseases. Therefore, further research is needed to determine the potential role of allicin in providing the desired effects in poultry production, and the optimization of the dosage regimens of this compound may be needed to ensure animal health.

## 5. Conclusions

The broad range of allicin’s biological properties provides the basis for its potential use as an alternative to antibiotic growth promoters currently banned in livestock farming. Among the many benefits of allicin in animal husbandry, particularly in poultry, its modulating effect on the intestinal microbiota, which includes the anaerobic spore-forming bacteria of the *Clostridium* spp. genus, appears to be important. Our study demonstrates the potential effect of allicin on the growth of this anaerobic microbiota isolated from the digestive tracts of quails. Based on the results of this study, it is noted that allicin supplied to animals in an appropriate dose not only reduces the level of *Clostridium* spp. in the digestive tract of animals in a statistically significant manner, but may also have a beneficial effect on modulating the species composition of the intestinal microbiome. Although the isolated strains belonged mainly to saprophytic species, some of them may be the etiological agent of many gastrointestinal diseases. At present, however, there are insufficient literature data available indicating a positive effect of this compound on anaerobic *Clostridium* spp. bacteria, and the total mechanism of action of allicin and its benefits are not yet fully understood. Given the above, it is necessary to conduct further research to determine the potential use of allicin in poultry farming so as to ensure the safety of animals at an appropriate level.

## Figures and Tables

**Table 1:** Results of the 16S rDNA analysis (see File S4 for further details of the isolates).

The Group from Which the Strain Was Isolated	Sequencing Results According to BLAST Analysis	% Similarity	Accession Number
G1–G4	*Clostridium* *sporogenes*	89–97%	CP082942.1
G1–G4		92%	CP013242.1
G1–G4		90–96%	DQ278864.1
G1–G4		95%	CP084367.1
G1–G4		94–95%	MT356160.1
G1–G4		94%	CP011663.1
G1	*Clostridium* *sartagoforme*	87%	OP862452.1
G1		93%	MN646980.1
G4		95%	MW450913.1
G3	*Clostridium butyricum*	94%	AY540106.1
G2	*Clostridium intestinale*	79%	MK559547.1
G2	*Clostridium perfringens*	94–96%	ON870866.1
G3		95–96%	ON870867.1
G2	*Clostridium* *saccharolyticum*	94%	CP002109.1
G4		95%	FJ957875.1

## Data Availability

Data are contained within the article and the Supplementary Materials.

## Supplementary Materials

The following supporting information can be downloaded at: https://www.mdpi.com/article/10.3390/ani15070906/s1, File S1: Feed components, Table S1: Composition and nutrient content of birds diet; File S2: Primers and probes sequences, Table S2: The sequences of primers and probe used to carry out the real-time PCR reaction for detection of the *ntnh* gene (Raphael and Andreadis, 2007, [21]); Table S3. The sequences of primers used to carry out the multiplex PCR reactions for the detection of genes that determine the occurence of particular toxotypes of *C. perfringens* species (Rood et al. 2018, [22]); Table S4: The sequences of primers used to carry out the PCR reactions that allows identifications of isolated strains at the species level (Vaneechoutte et al., 1996, [23]); File S3: The level of *Clostridium* spp. contamination depending on the group of animals tested and statistical analysis results; File S4: Detailed information on the 16S rDNA analysis results, Table S5: Results of the 16S rDNA Analysis.
