# Supplementary material for: The Impact of Allicin on the Growth of Clostridium spp. in the Digestive Track of Quails
Source: Animals (Basel). 2025 Mar 21;15(7):906. doi: 10.3390/ani15070906 (PMC11988147; doi:10.3390/ani15070906)
Supplement: Supplementary file 1 [file animals-15-00906-s001.zip › File S1. Feed components.pdf]

**Table S1.** Composition and nutrient content of birds diet.

| Ingredients | % | Nutrients composition calculated | |
|---|---|---|---|
| Soybean meal | 30.4 | Metabolizable energy [kcal/kg] | 2800 |
| Wheat | 27.15 | Crude protein | 21 |
| Corn | 15 | Crude fibre | 2.954 |
| Triticale | 10 | Arginine | 1.396 |
| Limestone | 5.18 | Lysine | 1.15 |
| Wheat bran | 5 | Methionine | 0.384 |
| Soybean oil | 4.3 | Methionine + cysteine | 0.75 |
| Monocalcium phosphate | 1.95 | Treonine | 0.78 |
| sodium chloride | 0.36 | Calcium | 2.5 |
| DL-Methionine | 0.08 | Total P | 0.858 |
| L-Lysine | 0.08 | Available P | 0.55 |
| Vitamin-minera premix* | 0.5 | Na | 0.16 |

* The premix provided per 1kg of diet: Vitamin A, 10000IU; vitamin D3, 2500IU; Vitamin E, 20 mg; 6-phytase, 150 FTU; Endo-1,4-beta-xylanase, 2000 IU
