# Supplementary material for: The Impact of Allicin on the Growth of Clostridium spp. in the Digestive Track of Quails
Source: Animals (Basel). 2025 Mar 21;15(7):906. doi: 10.3390/ani15070906 (PMC11988147; doi:10.3390/ani15070906)
Supplement: Supplementary file 1 [file animals-15-00906-s001.zip › File S2. Primers and probes sequences.pdf]

Table S2. The sequences of primers and probe used to carry out the real - time PCR reaction for detection of the *ntnh* gene (Raphael i Andreadis, 2007).

Primers	Sequences
F1	5′GATTTAAGTGAAAATTTATTTAATATAT′3
F2	5′CCACTAAATGATTTAAATGAA′3
F3	5′TGATGAAATACCTAATAGTATGTTAAAT′3
CD2F	5′GACATATCAGATAGTTTATTGGGA′3
R1	5′TTTAGCCATACAAATTAAATC′3
R2	5′ACTAGCCATACAAATTAGATC′3
R3	5′TATTAAACTTTCTTGAGCTA′3
**Probe**	**Sequence**
NTNH410	5′FAM-ATCAATGGTGGACACAATATTATAGTCA-BHQ′3

Table S3. The sequences of primers used to carry out the multiplex PCR reactions for the detection of genes that determine the occurence of particular toxotypes of *C. perfringens* species (Rood et al. 2018).

Genes	Primers	Sequences
netB	JRP6656	5′CTTCTAGTGATACCGCTTCAC′3
	JRP6655	5′CGTTATATTCACTTGTTGACGAAAG′3
etx	JRP4234	5′CCACTTACTTGTCCTACTAAC′3
	JRP4235	5′GCGGTGATATCCATCTATTC′3
iap	JRP5507	5′GGAAAAGAAAATTATAGTGATTGG′3
	JRP5508	5′CCTGCATAACCTGGAATGGC′3
plc	JRP4232	5′GCTAATGTTACTGCCGTTGACC′3
	JRP4233	5′CCTCTGATACATCGTGTAAG′3

cpe	JRP5179<br>JRP5180	5′GGAGATGGTTGGATATTAGG′3<br>5′GGACCAGCAGTTGTAGATA′3
cpb	JRP5181<br>JRP5182	5′GCGAATATGCTGAATCATCTA′3<br>5′GCAGGAACATTAGTATATCTTC′3

Table S4. The sequences of primers used to carry out the PCR reactions that allows identifications of isolated strains at the species level (Vaneechoutte et al., 1996).

Primers	Sequences
16S fw	5′TGGCTCAGATTGAACGCTGGCGGC3′
16S rev	5′TACCTTGTTACGACTTCACCCCA3′
