# Supplementary material for: The Impact of Allicin on the Growth of Clostridium spp. in the Digestive Track of Quails
Source: Animals (Basel). 2025 Mar 21;15(7):906. doi: 10.3390/ani15070906 (PMC11988147; doi:10.3390/ani15070906)
Supplement: Supplementary file 1 [file animals-15-00906-s001.zip › File S4. Detailed information on the 16S rDNA analysis results.pdf]

Table S5. Results of the 16S rDNA Analysis

**Amplicon sample**	**Sequence ID**	**% Similarity**	**Similar sequence from the GenBank database**
1	CP082942.1	95%	*Clostridium sporogenes* strain FDAARGOS_1471
2	CP082942.1	89%	*Clostridium sporogenes* strain FDAARGOS_1471
6	OP862452.1	87%	*Clostridium sartagoforme* strain NR_13-4
7	MN646980.1	93%	*Clostridium sartagoforme* strain CBA7517
8	CP082942.1	92%	*Clostridium sporogenes* strain FDAARGOS_1471
9	CP082942.1	93%	*Clostridium sporogenes* strain FDAARGOS_1471
13	CP013242.1	92%	Clostridium sporogenes strain CDC_67071
17	CP082942.1	96%	*Clostridium sporogenes* strain FDAARGOS_1471
18	CP082942.1	95%	*Clostridium sporogenes* strain FDAARGOS_1471
25	MK559547.1	79%	*Clostridium intestinale* strain B11
26	CP082942.1	97%	*Clostridium sporogenes* strain FDAARGOS_1471
27	FJ957875.1	95%	*Clostridium saccharolyticum* strain JPL_23
28	DQ278864.1	96%	*Clostridium sporogenes* isolate 152R-4
29	CP082942.1	93%	*Clostridium sporogenes* strain FDAARGOS_1471
30	ON870866.1	94%	*Clostridium perfringens* strain TMPC 3F511
31	CP084367.1	95%	*Clostridium sporogenes* strain FDAARGOS_1531
32	ON870867.1	96%	*Clostridium perfringens* strain TMPC 3S113
33	MT356160.1	94%	*Clostridium sporogenes* strain SM5
35	CP011663.1	94%	*Clostridium sporogenes* strain DSM 795
36	AY540106.1	94%	*Clostridium butyricum* strain CGS2
37	CP082942.1	96%	*Clostridium sporogenes* strain FDAARGOS_1471

38	ON870866.1	96%	*Clostridium perfringens* strain TMPC 3F511
39	ON870866.1	96%	*Clostridium perfringens* strain TMPC 3F511
40	CP082942.1	94%	*Clostridium sporogenes* strain FDAARGOS_1471
42	CP082942.1	95%	*Clostridium sporogenes* strain FDAARGOS_1471
43	MT356160.1	95%	*Clostridium sporogenes* strain SM5
44	CP002109.1	94%	*Clostridium saccharolyticum* WM1
45	ON870867.1	95%	*Clostridium perfringens* strain TMPC 3S113
46	DQ278864.1	95%	*Clostridium sporogenes* isolate 152R-4
47	MW450913.1	95%	*Clostridium sartagoforme* strain XN-T4
48	CP082942.1	95%	*Clostridium sporogenes* strain FDAARGOS_1471
49	CP082942.1	94%	*Clostridium sporogenes* strain FDAARGOS_1471
50	DQ278864.1	90%	*Clostridium sporogenes* isolate 152R-4
